# Long-Term Effectiveness of a Mobile-Based Breastfeeding Program for Women with Gestational Diabetes: 6-Month Follow-Up of a Quasi-Experimental Study

**DOI:** 10.3390/healthcare14070917

**Published:** 2026-04-01

**Authors:** Seungmi Park, Young Mi Ryu, Eunju Kwak

**Affiliations:** 1 Department of Nursing Science, Research Institute of Nursing Science, College of Nursing, Chungbuk National University, Cheongju 28644, Republic of Korea; spark2020@chungbuk.ac.kr; 2 Department of Nursing Science, Baekseok University, Cheonan 31065, Republic of Korea; youngmiryu@bu.ac.kr; 3 Department of Nursing, Research Institute for Basic Sciences, College of Bio-Health, Hoseo University, Asan 31499, Republic of Korea

**Keywords:** breastfeeding, gestational diabetes, mobile health, IMB model, health promotion, mHealth, mobile application

## Abstract

**Highlights:**

**What are the main findings?**

- A mobile-based breastfeeding promotion program for gestational diabetes melitus(M-BFGDM) effectively improved and maintained breastfeeding knowledge among mothers with gestational diabetes mellitus (GDM) up to 6 months postpartum.
- Despite cognitive improvements, knowledge alone was insufficient to sustain long-term breastfeeding practices due to persistent physical barriers.

**What are the implications of the main findings?**

- Sustained breastfeeding practice for mothers with GDM requires intensive, individualized clinical support to address specific issues such as low milk supply and latching difficulties.
- Digital health interventions should shift to a ‘hybrid support’ model, integrating mobile platforms with existing healthcare systems to trigger immediate, hands-on lactation support.

**Abstract:**

Background: Mothers with gestational diabetes mellitus (GDM) face specific challenges in breastfeeding, yet data on the long-term effectiveness of mobile-based interventions remain limited. This study aimed to evaluate the long-term effectiveness of a Mobile-Based Breastfeeding Promotion Program for GDM (M-BFGDM) on breastfeeding knowledge, self-efficacy, and practice rates up to 6 months postpartum. Methods: A nonequivalent control group pretest–posttest quasi-experimental study was conducted. Participants were recruited from an online community. The intervention group received the M-BFGDM, which included Information–Motivation–Behavioral Skills (IMB) model-based educational videos and KakaoTalk counseling. Data were collected at prenatal, 1-week, 1-month, and 6-month postpartum time points. Data were analyzed using generalized estimating equations (GEE) and repeated-measures ANOVA. Results: The final analysis included 38 participants (experimental group, *n* = 18; control group, *n* = 20). The M-BFGDM was effective in improving breastfeeding knowledge among women with GDM (*p* = 0.003). However, the intervention did not significantly influence the trajectory of breastfeeding self-efficacy or prevent the decline in practice rates over 6 months compared to the control group. Conclusions: These findings suggest that while mobile education enhances knowledge, sustained breastfeeding requires more intensive, individualized support to address physical barriers, such as low milk supply and latch difficulties.

## 1. Introduction

### 1.1. Current Status and Importance of Gestational Diabetes Mellitus (GDM)

Gestational diabetes mellitus (GDM) is a metabolic condition first diagnosed during pregnancy that poses significant health risks to both mothers and their babies. According to the International Diabetes Federation [1], around 16.7% of pregnant women globally had hyperglycemia during pregnancy, of which 80.3% were classified as GDM. The increasing global burden of GDM highlights the urgency of effective management strategies during and after pregnancy.

In South Korea, the prevalence of GDM has shown a steady upward trend, largely driven by rising maternal age. According to the Korean Statistical Information Service [2], the prevalence rose from 1.7–3.9% in the early 2000s to 10.8% in 2023. The proportion of mothers aged 35 or older also increased significantly, from 36.9 per 1000 in 2010 to 49.6 per 1000 in 2020. This demographic shift is a key contributor to the growing burden of GDM.

GDM increases the risk of neonatal complications such as macrosomia, hypoglycemia, and preterm birth. It also poses long-term health risks to mothers, including preeclampsia, hypertension, and a higher likelihood of developing type 2 diabetes [3]. Consequently, it is essential to establish effective prenatal and postpartum management strategies.

Mothers diagnosed with GDM require highly specialized attention, as metabolic complications during pregnancy can exacerbate the risk of severe maternal morbidity [4]. Furthermore, they face a substantially elevated lifelong risk of developing type 2 diabetes, a progression influenced by various genetic and physiological risk factors [5].

### 1.2. Importance of Breastfeeding and the Current Status Among Mothers with GDM

Breastfeeding is the most beneficial source of nutrition for newborns and infants. Breastfed children show lower incidences of respiratory and gastrointestinal diseases, constipation, eczema, and allergies than formula-fed infants, and tend to have greater emotional stability [6]. The World Health Organization (WHO) and United Nations Children’s Emergency Fund (UNICEF) recommend exclusive breastfeeding for at least the first six months to ensure balanced health and development.

According to the 2021 Family and Birth Survey by the Korea Institute for Health and Social Affairs, the exclusive breastfeeding rate increased from 26.0% in the first week to 45.3% by the third week but sharply declined to 5.2% by six months [7]. Although breastfeeding among mothers with GDM is effective in regulating postpartum blood glucose and reducing the risk of type 2 diabetes [8], their rates of exclusive breastfeeding for more than six months are significantly lower compared to non-GDM mothers [9,10].

Clinically, mothers with GDM face unique physiological barriers that hinder breastfeeding initiation and continuation. Maternal insulin resistance and poor glycemic control can delay lactogenesis stage II, often prolonging the onset of copious milk secretion. Furthermore, potential alterations in breast milk composition and infant factors, such as macrosomia or neonatal hypoglycemia, frequently necessitate early formula supplementation. These intertwined physiological and clinical challenges underscore the need for early, targeted, and continuous lactation support tailored specifically for this population.

Barriers to breastfeeding among mothers with GDM include physical discomfort after cesarean section, delayed lactogenesis, misconceptions about transmitting diabetes through breast milk, and sociocultural pressures related to infant feeding. These complex factors underscore the need for evidence-based, practical education and support interventions [11].

### 1.3. Duration of Breastfeeding and Its Role in Preventing Metabolic Disorders

Breastfeeding for more prolonged durations has been associated with a lower risk of metabolic disorders. Specifically, breastfeeding beyond one month reduces the risk by approximately 26%, and beyond one year by up to 36% [12]. This protective effect is believed to result from hormonal and metabolic changes during lactation, including improved insulin sensitivity, mobilization of fat stores, and reduced visceral adiposity. Ziegler et al. [13] further found that extended breastfeeding significantly lowers the risk of type 2 diabetes among women with a history of GDM, potentially by preserving β-cell function and reducing postnatal glucose intolerance.

### 1.4. Need for Mobile-Based Breastfeeding Promotion Programs

Mobile health platforms offer an effective way to deliver personalized health education without time or location constraints. Compared with conventional methods, they enhance user autonomy, interactivity, and real-time accessibility. Moreover, prior research indicates that interventions combining education with emotional and informational support are more effective in sustaining breastfeeding than education alone [14]. Also, community- and home-based programs have successfully improved breastfeeding rates for up to 12 months postpartum [15]. Nevertheless, most mobile interventions remain limited, as they lack GDM-specific tailoring and theoretical grounding.

In response, the Mobile-Based Breastfeeding Program for Gestational Diabetes Mellitus (M-BFGDM) was developed using the Information-Motivation-Behavioral Skills (IMB) model to address specific lactation barriers faced by women with GDM, such as delayed lactogenesis and postpartum blood glucose instability. The program incorporated real-time interaction via KakaoTalk (Kakao Corp., Jeju, Republic of Korea), modular educational videos, and behavior-reinforcing feedback to promote sustained engagement and individualized care. This design ensures technical and conceptual differentiation from general mobile health programs.

### 1.5. Need and Purpose of the Study

Most previous studies on breastfeeding interventions have concentrated on short-term outcomes during pregnancy or the immediate postpartum period, providing limited evidence of sustained effectiveness. Kwak and Park [16] developed the M-BFGDM and evaluated its effects from prenatal care through one week and one month postpartum. This study is a follow-up to their work, extending the evaluation period to six months postpartum to examine long-term outcomes. The purpose is to determine whether the M-BFGDM has lasting effects on breastfeeding behaviors among women with GDM. Ultimately, this study seeks to verify the program’s effectiveness in sustaining breastfeeding practices and improving maternal and infant health outcomes in this high-risk population.

The specific objectives of this study are to evaluate the effects of the M-BFGDM on breastfeeding knowledge, intention, self-efficacy, methods, and practice rate at six months postpartum among women with GDM.

## 2. Materials and Methods

### 2.1. Study Design

This study employed a nonequivalent control group pretest-posttest quasi-experimental design to evaluate the long-term effectiveness of a mobile-based breastfeeding promotion program tailored for women diagnosed with gestational diabetes mellitus (GDM). The study protocol adhered to the TREND (Transparent Reporting of Evaluations with Nonrandomized Designs) statement for nonrandomized evaluations.

Because allocation concealment and randomization were not feasible in this pragmatic clinical setting, participants were assigned to the intervention or control group using a sequential recruitment method. To minimize contamination (diffusion of intervention effects), the two groups were recruited in separate time windows: the control group was recruited first (4 March 2022–31 May 2022) to establish baseline standard care outcomes. After the completion of control group enrollment and assessments, the intervention group was subsequently recruited (1 July 2022–30 August 2022). This sequential design ensured that participants in the control group were not exposed to the mobile intervention content or interactions intended for the experimental group. The study was registered with the Clinical Research Information Service (CRIS) under the Korea Disease Control and Prevention Agency (CRIS Registration No: KCT0007818).

### 2.2. Participants

This study recruited participants using convenience sampling from an online community for women with GDM hosted on Naver. Eligibility criteria included being 20 years or older, having a confirmed diagnosis of GDM during pregnancy, and providing informed consent with a complete understanding of the study purpose.

The age criterion was established to exclude teenage pregnancies, which are associated with distinct psychosocial and developmental variables that could confound the results. Additionally, women diagnosed with pre-gestational diabetes (Type 1 or Type 2) or those suffering from other severe chronic diseases were strictly excluded to isolate the specific impact of GDM on breastfeeding outcomes.

This study shares its cohort with a previous study by Kwak and Park [16]. While the initial study evaluated outcomes up to one month postpartum, this study extends the follow-up to six months postpartum and uses generalized estimating equations (GEE) for longitudinal analysis to demonstrate the intervention’s long-term impact.

### 2.3. Intervention

The M-BFGDM was developed using the ADDIE model (Analysis, Design, Development, Implementation, Evaluation) and grounded in the Information-Motivation-Behavioral Skills (IMB) model. Specifically, in the Analysis phase, a needs assessment identified the distinct physical and psychosocial lactation barriers faced by mothers with GDM.

During the Design and Development phases, these specific needs were mapped onto the IMB constructs to produce modular educational videos and structured KakaoTalk counseling protocols. In the Implementation phase, the intervention was delivered dynamically from the prenatal period up to 6 months postpartum. Finally, the Evaluation phase involved the longitudinal assessment of breastfeeding knowledge, self-efficacy, and practice rates. To systematically address the specific barriers faced by mothers with GDM, the intervention was explicitly mapped to the IMB constructs and linked to the study outcomes.

First, the Information component aimed to improve ‘breastfeeding knowledge’ through modular educational videos covering GDM-specific lactation physiology and milk characteristics. Second, the Motivation component was designed to enhance ‘breastfeeding self-efficacy’ through individualized 1:1 KakaoTalk counseling, providing emotional support and emphasizing the protective metabolic benefits for both the mother and the infant. Finally, the Behavioral Skills component directly targeted the ‘breastfeeding practice rate’ by offering actionable training on latching techniques, breast massage (e.g., Oketani method), and pumping strategies, supplemented by real-time troubleshooting for physical challenges such as low milk supply or nipple pain.

The program uniquely addressed GDM-specific barriers, such as delayed lactogenesis and postpartum glucose instability, differentiating it from general breastfeeding apps.

**Figure 1:**
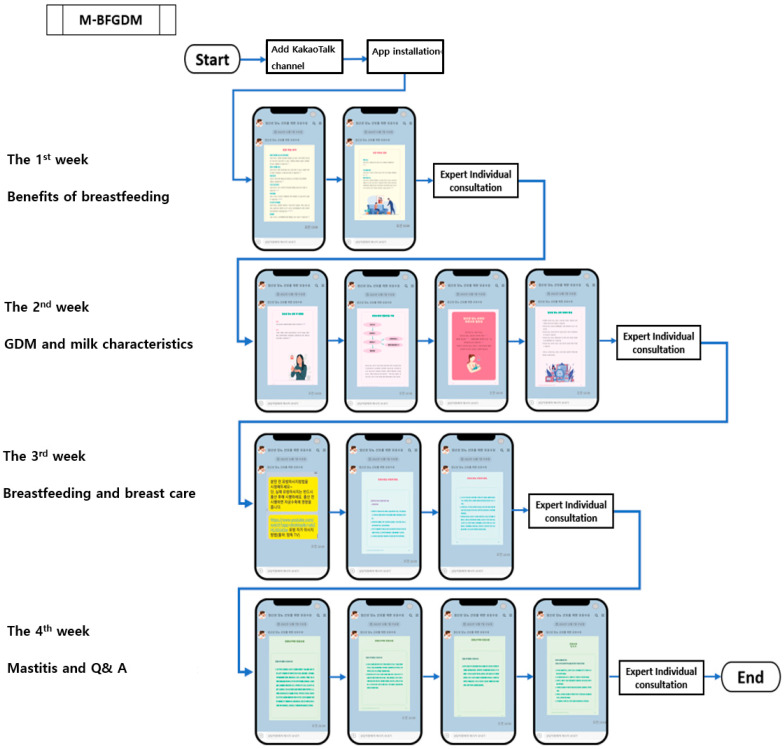
User flow diagram of the Mobile-Based Breastfeeding Promotion Program for GDM (M-BFGDM).

Delivered via a KakaoTalk channel, the intervention provided weekly educational videos and personalized breastfeeding guidance during the prenatal and postpartum periods (Table 1). Figure 1 shows the step-by-step user journey, including KakaoTalk notifications, access to modular educational videos, and initiation of one-on-one counseling.

The program incorporated real-time interaction via KakaoTalk, modular educational videos, and behavior-reinforcing feedback to promote sustained engagement. To enhance reproducibility, the intervention components, dose, tailoring rules, and fidelity indicators are specified in Appendix A in a TIDieR-aligned format.

### 2.4. Measures

To maintain methodological consistency with the initial study, the same validated instruments were used as follows:

General Characteristics: Age, education level, delivery method, prior birth experience, economic status, employment type, postpartum caregiver type, diabetes medication use, and stress level (assessed using the Numeric Rating Scale) were collected.

Breastfeeding Knowledge: A 14-item questionnaire measured mothers’ knowledge about breastfeeding in the context of GDM. Each correct response received one point, with total scores ranging from 0 to 14. The initial study reported a Kuder–Richardson 20 of 0.91. In the present study, the Kuder–Richardson 20 (KR-20) for this instrument was 0.92.

Breastfeeding Self-Efficacy: A 12-item, 5-point Likert scale assessed participants’ confidence in breastfeeding practices, with higher scores indicating stronger self-efficacy (Cronbach’s α = 0.94 in the initial study). In the current sample, the Cronbach’s α was 0.94.

Breastfeeding Practice Rate: Breastfeeding status was classified based on the WHO’s Infant and Young Child Feeding Classification. Levels 1 (exclusive breastfeeding) and 2 (breastfeeding > formula) were defined as “breastfeeding practice” (score 1), while other levels were scored as 0.

Reasons for Breastfeeding Discontinuation: For participants who reported stopping breastfeeding or transitioning to formula feeding during the postpartum follow-up periods, an open-ended question was included in the survey to explore their specific reasons and physical barriers leading to early cessation.

### 2.5. Data Collection

Data were collected via structured web-based surveys distributed through secure URL links at four time points: during pregnancy (baseline), and at 1 week, 1 month, and 6 months postpartum.

The 6-month postpartum follow-up data were collected online using Google Forms (Google LLC, Mountain View, CA, USA) from 1 October to 31 December 2024. Participants were contacted through pre-established communication channels and KakaoTalk to encourage survey completion. During this follow-up period, 4 participants from the experimental group and 5 from the control group were lost to follow-up due to contact loss.

### 2.6. Statistical Analysis

Data were analyzed using SPSS 26.0 (IBM Corp., Armonk, NY, USA) and SAS software (SAS Institute Inc., Cary, NC, USA). General characteristics were analyzed using frequencies, percentages, means, and standard deviations. Homogeneity between groups was tested using independent *t*-tests, Mann–Whitney U test, χ^2^ tests, and Fisher’s exact tests.

To assess the program’s long-term effectiveness, changes in breastfeeding knowledge, self-efficacy, and practice rates across the four time points (prenatal, 1 week, 1 month, and 6 months) were analyzed. To ensure appropriate analysis based on data characteristics, a repeated-measures ANOVA was used for continuous variables that met the assumption of normality (i.e., breastfeeding self-efficacy). Conversely, Generalized Estimating Equations (GEE) were utilized for data that did not meet normality assumptions (i.e., breastfeeding knowledge) and for binary outcomes (i.e., breastfeeding practice rate), as GEE is robust against non-normal distributions and effectively handles correlated repeated measures over time. To provide a clearer interpretation of the intervention’s practical significance, effect sizes (e.g., Cohen’s d for continuous variables and Odds Ratios [OR] for binary outcomes) and 95% Confidence Intervals (CIs) were calculated and reported alongside *p*-values. Additionally, cross-tabulation (Chi-square analysis) was performed to compare breastfeeding practice rates between the two groups at each specific time point (1 week, 1 month, and 6 months).

Primary analyses used an available-case approach without imputation. Given the risk of attrition in nonrandomized longitudinal studies, sensitivity checks were performed using complete-case analyses to assess the robustness of primary inferences. A statistical significance level of *p* < 0.05 was applied.

Qualitative responses regarding the reasons for breastfeeding discontinuation were categorized based on thematic similarities (e.g., insufficient milk supply, breast problems) and summarized descriptively.

### 2.7. Ethical Considerations

This study complied with the ethical guidelines established in previous research. To ensure the ethical protection of participants, approval was obtained from the Institutional Review Board (IRB) of Chungbuk National University before the start of the study (IRB No. CBNU-202402-HR-0468).

During the preparation of this work, the authors used ChatGPT (OpenAI, San Francisco, CA, USA) solely for language editing and formatting in accordance with journal guidelines. Generative AI was not used to generate scientific hypotheses, analyze data, or draft the initial manuscript.

## 3. Results

**Figure 2:**
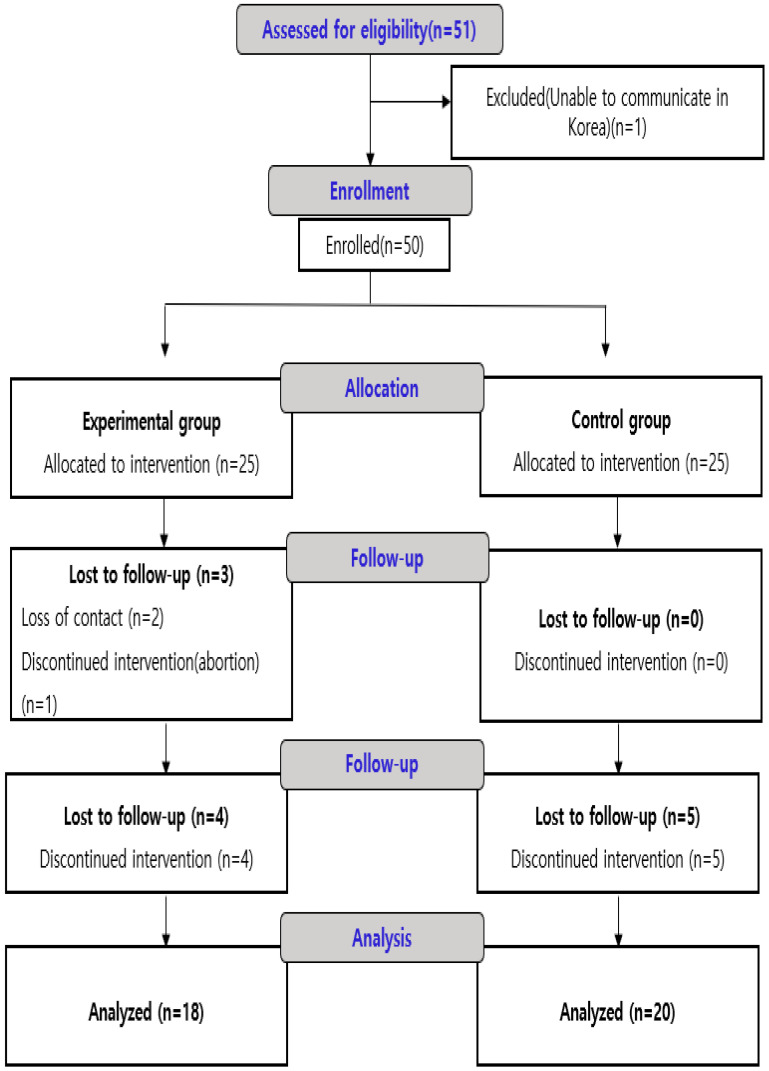
CONSORT flow diagram of the participants’ enrollment.

A total of 51 participants were initially recruited. One participant was excluded due to an inability to communicate in Korean, resulting in 50 enrolled participants (control group *n* = 25; experimental group *n* = 25). During the 6-month follow-up survey window (1 October–31 December 2024), 4 participants in the intervention group and 5 in the control group were lost to follow-up due to uncontactable status. The final analysis included 38 participants (control group n = 20; experimental group n = 18) (Figure 2).

### 3.1. General Characteristics and Baseline Homogeneity

Table 2 presents the general characteristics of the participants. There were no statistically significant differences between the intervention and control groups regarding age, education level, delivery method, economic status, or employment type (*p* > 0.05), confirming baseline homogeneity. Furthermore, baseline scores for breastfeeding knowledge and self-efficacy did not differ significantly between groups (Table 2).

To assess potential bias due to participant dropout, an attrition analysis was conducted comparing the baseline characteristics of completers (*n* = 38) and non-completers (*n* = 9). There were no statistically significant differences between the two groups regarding age, education level, or delivery method (all *p* > 0.05), indicating that attrition occurred at random and did not systematically bias the final analytic sample.

### 3.2. Outcomes of the M-BFGDM Program

#### 3.2.1. Breastfeeding Knowledge

Since the data did not meet normality assumptions, Generalized Estimating Equations (GEE) were used. There was no significant interaction effect between time and group (χ^2^ = 2.01, *p* = 0.570). However, there were significant main effects for group (χ^2^ = 8.69, *p* = 0.003, Cohen’s d = 1.02, 95% CI [0.33, 1.70]) and time (χ^2^ = 13.32, *p* = 0.004, Cohen’s d = 1.28, 95% CI [0.55, 1.98]) (Table 3).

Based on Cohen’s criteria, these values indicate a large effect size, suggesting substantial practical and clinical improvement in breastfeeding knowledge attributable to the intervention.

#### 3.2.2. Breastfeeding Self-Efficacy

Repeated measures ANOVA was used. The assumption of sphericity was met. There was no significant interaction effect between time and group (F = 0.19, *p* = 0.859), nor were there significant main effects for group (F = 0.03, *p* = 0.863) or time (F = 0.60, *p* = 0.961) (Table 3).

#### 3.2.3. Breastfeeding Practice Rate

Longitudinal analysis using the GEE model showed no significant interaction between time and group over the 6-month period (χ^2^ = 0.10, *p* = 0.746). However, there were significant overall main effects for group (χ^2^ = 143.97, *p* < 0.001, OR = 0.89, 95% CI [0.11, 7.06]) and time (χ^2^ = 17.78, *p* < 0.001) (Table 3). Conversely, cross-tabulation analysis conducted at each specific assessment point revealed no significant differences in exclusive breastfeeding practice rates between the groups at 1 week (χ^2^ = 0.01, *p* =1.000), 1 month (χ^2^ = 1.90, *p* = 0.488), and 6 months postpartum (χ^2^ = 0.63, *p* = 0.327) (Table 3).

#### 3.2.4. Reasons for Breastfeeding Discontinuation

Participants who transitioned to formula feeding cited specific barriers (Table 4). The primary reasons for discontinuation included insufficient breast milk supply, breast problems (e.g., sore nipples, pain), and difficulties with breastfeeding techniques (e.g., latching issues). Qualitative feedback highlighted that physical challenges often led to early cessation despite initial intentions to breastfeed (Table 4).

## 4. Discussion

### 4.1. Principal Findings

In this study, the M-BFGDM effectively enhanced breastfeeding knowledge among women with GDM, maintaining higher scores than the control group through 6 months postpartum. However, despite this increase in knowledge, the intervention did not significantly improve breastfeeding self-efficacy or prevent the decline in breastfeeding practice rates over the 6-month period.

In our data, improved knowledge did not translate into sustained breastfeeding behavior, indicating that information alone may be insufficient in this population. This discrepancy underscores a critical clinical reality: cognitive improvements cannot easily override deeply rooted structural and physical barriers. As reflected in our qualitative data, despite possessing adequate knowledge, mothers with GDM confronted insurmountable physical challenges such as persistently low milk supply, severe infant latching difficulties, and breast pain. Furthermore, the lack of an integrated healthcare system support network to provide immediate, hands-on intervention when these physical difficulties first arose likely contributed to the rapid transition to formula feeding.

Notably, the divergence between improved knowledge and declining breastfeeding practice represents a clinically meaningful gap that may not be apparent when only educational outcomes are considered. The sharp decline in practice rates in both groups indicates that knowledge does not automatically translate into sustained practice, especially when mothers face physical challenges. As noted in prior research, sufficient education must be paired with professional preparation and support to retain breastfeeding knowledge [17].

### 4.2. Comparison with Prior Work

The decline in breastfeeding rates observed in our study mirrors global and national trends. Worldwide, only 38% of infants are exclusively breastfed for the first 6 months [18]. In South Korea, the drop-off is even more precipitous, with exclusive breastfeeding rates falling to 5.6% at 6 months and to roughly 1% at 12 months [19].

Common barriers reported by our participants, such as breast pain and social challenges, are consistent with those identified in previous studies [20,21]. However, mothers with GDM face additional physiological hurdles. Delayed lactogenesis, altered breast milk composition, and insulin resistance can impair milk production and exacerbate breastfeeding difficulties [22,23]. Longitudinal studies have confirmed that GDM is a significant risk factor for early cessation; for instance, Vietnamese mothers with GDM were found to be 1.39 times more likely to stop breastfeeding early than healthy mothers [9,10,24].

To overcome these barriers, recent evidence suggests that “contactless” education must be supplemented with active clinical support. A recent Delphi study reached a consensus that breastfeeding preparedness requires a combination of education, support, and practical skills training (e.g., nipple care, latching techniques) [25]. Furthermore, randomized controlled trials demonstrate that proactive peer and professional coaching significantly prolongs breastfeeding duration and exclusivity [26,27]. Motivational interviewing-based coaching has also been shown to reduce postpartum depression and extend breastfeeding [28]. A Cochrane review further confirmed that structured, proactive coaching provided across 4–8 contact points consistently reduces early cessation [29]. In our study, many mothers with GDM continued to report practical difficulties, such as pain, latching problems, and low milk supply, despite improved knowledge, suggesting that educational content alone may be insufficient without timely practical support.

### 4.3. Strengths and Clinical Implications

A major strength of this study is its longitudinal evaluation extending up to six months postpartum. By tracking a high-risk population (mothers with GDM) over a prolonged period, this study captures the long-term trajectory of breastfeeding behaviors, revealing the critical drop-off points that short-term studies often miss. The findings offer vital implications for clinical practice and digital health strategies. The implementation of mHealth programs in other contexts or locations should shift from a purely educational paradigm to a ‘hybrid support’ model. In practice, mobile platforms should be used not only to disseminate information but also as an early warning system. When a mother reports latching difficulties or low milk supply via the app, it should automatically trigger coordinated, personalized lactation support from clinical services (e.g., an in-person visit from a lactation consultant or a telehealth physical assessment). Integrating mobile health interventions directly with local healthcare systems will bridge the gap between knowing how to breastfeed and physically being able to sustain it.

### 4.4. Limitations

This study has several limitations. First, it utilized a quasi-experimental design with a small sample size (n = 38) and convenience sampling, which limits causal inference and generalizability. The use of a sequential recruitment strategy—while necessary to prevent contamination of the intervention within the same online community—introduces potential temporal bias, as external factors occurring between the two recruitment periods could have influenced the outcomes. Furthermore, the absence of randomization raises the possibility of selection bias and residual confounding. Second, we did not conduct an a priori sample size calculation. A post hoc power analysis revealed that the statistical power for detecting differences in breastfeeding self-efficacy (time × group interaction) was 0.096, which is far below the conventional threshold of 0.80. Therefore, the non-significant findings for this variable should be interpreted with caution, as the small sample size and resulting lack of statistical power likely limited our ability to detect the true long-term effects of the intervention. Third, the relatively high attrition rate during the 6-month follow-up, though common in longitudinal digital health studies due to postpartum maternal exhaustion, further reduced statistical power. Finally, prior breastfeeding experience was not measured as a separate baseline variable, which may have limited our ability to fully account for its potential confounding effect on long-term breastfeeding practices.

### 4.5. Future Research Directions

This study shows that mobile-based education can improve women with GDM’s breastfeeding knowledge, but knowledge gains alone were insufficient to sustain breastfeeding practice over time. The findings highlight that mothers often need practical, individualized support when physical difficulties arise during the postpartum period.

Future intervention models should therefore combine educational delivery with clinically guided follow-up and be evaluated in larger randomized studies.

#### Enhance Personalized Clinical Follow-Up Support

Because participants frequently reported physical barriers during the postpartum period, individualized follow-up support may help mothers apply breastfeeding knowledge in daily practice.

1. Incorporate Breastfeeding Skills Training

Practical guidance focused on common early problems, such as latching difficulties and nipple pain, may help reduce early discontinuation.

2. Develop GDM-Specific Breastfeeding Guidelines

Clinical guidance tailored to the physiological characteristics of mothers with GDM may provide more consistent support across care settings.

3. Conduct Longitudinal Multi-Center Studies

Larger-scale and multi-center longitudinal studies are recommended to validate the generalizability and scalability of mobile-based interventions for mothers with GDM.

## 5. Conclusions

This study demonstrates that while the Mobile-Based Breastfeeding Promotion Program (M-BFGDM) effectively improved and maintained breastfeeding knowledge among mothers with gestational diabetes mellitus (GDM) up to six months postpartum, these cognitive improvements alone were insufficient to sustain long-term breastfeeding practices. Mothers with GDM face unique and persistent physical barriers, such as low milk supply and severe infant latching difficulties, which cannot be overcome solely through contactless digital education. Therefore, to ensure the long-term continuation of breastfeeding in this high-risk population, future digital health strategies must shift towards a ‘hybrid support’ model. Mobile health platforms should be deeply integrated with existing healthcare systems to serve as early-warning systems that trigger prompt, individualized, and hands-on clinical lactation support.

## Figures and Tables

**Table 1:** Intervention contents, tools, and methods by period.

Period	Intervention and Contents	Tools	Methods
Prenatal (4 weeks before)	Introduction to M-BFGDM	KakaoTalk channel	App usage guide
	Benefits of breastfeeding	Zoom (Zoom Video Communications, Inc., San Jose, CA, USA; 1:1)	Real-time video
Prenatal (3 weeks before)	GDM and milk characteristics	KakaoTalk channel	PDF resource
Prenatal (2 weeks before)	Breastfeeding and breast care	KakaoTalk channel	Educational video link
Prenatal (1 week before)	Mastitis and Q&A	KakaoTalk channel	PDF resource
Postpartum (up to 6 months postpartum)	Interactive support	KakaoTalk channel	1:1 Counseling

**Table 2:** Homogeneity test of the general characteristics and outcome variables between the two groups at baseline.

Characteristics	Categories	Exp. (*n* = 18)	Con. (*n* = 20)	χ^2^/t/Z	*p*
		*n* (%)	*n* (%)		
Age	≤34	8 (44.4)	11 (55.0)	0.42	0.746
	≥35	10 (55.6)	9 (45.0)		
Education	≤Junior college	3 (16.7)	6 (30.0)		0.454 †
	≥University	15 (83.3)	14 (70.0)		
Delivery method	Normal delivery	7 (38.9)	9 (45.0)	0.15	0.752
	Cesarean section	11 (61.1)	11 (55.0)		
Childbirth history	Yes	6 (33.3)	6 (30.0)	0.05	0.999
	No	12 (66.7)	14 (70.0)		
Economic status	Enough	2 (11.1)	4 (20.0)		0.663 †
	Not enough	16 (77.8)	16 (80.0)		
Occupational status	Full-time	8 (44.4)	8 (40.0)	0.92	0.516
	Part-time or housewife	10 (55.6)	12 (60.0)		
Parenting support	Family	16 (88.9)	19 (95.0)		0.595 †
	Not family	2 (11.1)	1 (5.0)		
Stress level (0–10)	(Mean ± SD)	5.17 ± 2.85	5.35 ± 1.73	−0.89 ‡	0.942
Breastfeeding knowledge		7.78 ± 4.50	5.35 ± 3.36	−1.67 ‡	0.099
Breastfeeding self-efficacy		2.89 ± 0.86	2.84 ± 0.69	0.20	0.840

Note. † Fisher’s exact test; ‡ Mann–Whitney U test; Exp. = Experimental group; Con. = Control group; SD = Standard deviation.

**Table 3:** Result of the generalized estimating equation model for breastfeeding knowledge, breastfeeding practice rate, the repeated-measures ANOVA for breastfeeding self-efficacy, and the chi-square for breastfeeding practice rate.

Variables	Time		Experimental Group (*n* = 18)	Control Group (*n* = 20)	Group Effect	Time Effect	Group × Time Effect
Breastfeeding knowledge	Prenatal		7.78 ± 4.51	5.35 ± 3.36	Wald χ^2^ = 8.69 (*p* = 0.003) †	Wald χ^2^ = 13.32 (*p* = 0.004) †	Wald χ^2^ = 2.01 (*p* = 0.570) †
	1 week postpartum		10.44 ± 2.20	6.40 ± 4.16			
	1 month postpartum		10.61 ± 2.54	6.30 ± 4.76			
	6 months postpartum		10.78 ± 2.92	7.30 ± 5.31			
Breastfeeding self-efficacy	Prenatal		2.89 ± 0.18	2.84 ± 0.17	F = 0.03 (*p* = 0.863)	F = 0.60 (*p* = 0.961)	F = 0.19 (*p* = 0.859)
	1 week postpartum		2.93 ± 0.21	2.77 ± 0.20			
	1 month postpartum		2.90 ± 0.26	2.91 ± 0.25			
	6 months postpartum		2.87 ± 0.25	2.88 ± 0.23			
Breastfeeding practice rate	Overall(GEE)				Wald χ^2^ = 143.97 (*p* < 0.001) †	Wald χ^2^ = 17.78 (*p* < 0.001) †	Wald χ^2^ = 0.10 (*p* = 0.746) †
	1 week postpartum	Yes	16 (88.9%)	18 (90.0%)	χ^2^ = 0.01 (*p* = 1.000) ‡		
		No	2 (11.1%)	2 (10.0%)			
	1 month postpartum	Yes	18 (100.0%)	18 (90.0%)	χ^2^ = 1.90 (*p* = 0.488) ‡		
		No	0 (0.0%)	2 (10.0%)			
	6 months postpartum	Yes	5 (27.8%)	8 (40.0%)	χ^2^ = 0.63 (*p* = 0.327) ‡		
		No	13 (72.2%)	12 (60.0%)			

Note. † Generalized Estimating Equations (GEE); ‡ Chi-square analysis (cross-tabulation at each time point).

**Table 4:** Reasons to stop breastfeeding.

Category	Specific Description
Insufficient breast milk supply	“Perhaps because the amount of breast milk was small and the baby was born small at 35 weeks and one day, the baby had no sucking power. Even after leaving the nursing center, the baby could only drink about 10 to 30 mL of formula, and the weight did not increase. I tried pumping and feeding directly but failed every time, so I just switched to formula feeding.” (A03) “I stopped breastfeeding because my milk supply did not increase from 40 mL.” (B01) “The amount of milk was significantly low, so I am feeding more formula than breast milk.” (B22) “I had a child through in vitro fertilization, and after the cesarean surgery, when I started pumping, my breasts became very sore, so I received Oketani massage about four times. The masseuse said that because I had undergone hormone treatment, my milk was condensing a lot due to the hormones, and my milk supply appeared low. I tried breastfeeding, but the amount was small, and I was physically too weak to care for the child while pumping consistently. I could not breastfeed as I had originally planned and ended up formula feeding.”(B14)
Breast problems	“The amount of milk was low, and my nipples were so sore when pumping that I failed to breastfeed.” (B04)
How to breastfeed	“Breastfeeding failed because the baby did not want to suckle.”(B02)

Note. A = Control group; B = Experimental group.

## Data Availability

The data that support the findings of this study are available on request from the corresponding author. The data are not publicly available due to privacy concerns.

## Abbreviations

The following abbreviations are used in this manuscript:

GDM	Gestational diabetes mellitus
IMB	Information–motivation–behavioral skills
M-BFGDM	Mobile-based breastfeeding promotion program for gestational diabetes mellitus
TREND	Transparent reporting of evaluations with nonrandomized designs
TIDieR	Template for intervention description and replication

## Appendix A

		**The TIDieR (Template for Intervention Description and Replication) Checklist:** Information to include when describing an intervention and the location of the information		
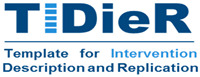
**Item number**	**Item**		**Where located**	
			Primary paper (page or appendix number)	Other (details)
	**BRIEF NAME**			
**1.**	Provide the name or a phrase that describes the intervention.		Page 4 (Section 2.3)	______________
	**WHY**			
**2.**	Describe any rationale, theory, or goal of the elements essential to the intervention.		Page 4 (Section 2.3)	_____________
	**WHAT**			
**3.**	Materials: Describe any physical or informational materials used in the intervention, including those provided to participants or used in intervention delivery or in training of intervention providers. Provide information on where the materials can be accessed (e.g., online appendix, URL).		Page 4 (Section 2.3, Table 1)	_____________
**4.**	Procedures: Describe each of the procedures, activities, and/or processes used in the intervention, including any enabling or support activities.		Pages 4–5 (Section 2.3, Table 1, Figure 1)	_____________
	**WHO PROVIDED**			
**5.**	For each category of intervention provider (e.g., psychologist, nursing assistant), describe their expertise, background and any specific training given.		Pages 4–5 (Section 2.3, Figure 1)	_____________
	**HOW**			
**6.**	Describe the modes of delivery (e.g., face-to-face or by some other mechanism, such as internet or telephone) of the intervention and whether it was provided individually or in a group.		Page 4 (Section 2.3, Table 1)	_____________
	**WHERE**			
**7.**	Describe the type(s) of location(s) where the intervention occurred, including any necessary infrastructure or relevant features.		Page 4 (Section 2.3)	_____________
	**WHEN and HOW MUCH**			
**8.**	Describe the number of times the intervention was delivered and over what period of time including the number of sessions, their schedule, and their duration, intensity or dose.		Page 4 (Section 2.3, Table 1)	_____________
	**TAILORING**			
**9.**	If the intervention was planned to be personalised, titrated or adapted, then describe what, why, when, and how.		Page 4 (Section 2.3, Table 1)	_____________
	MODIFICATIONS			
**10.**	If the intervention was modified during the course of the study, describe the changes (what, why, when, and how).		N/A	_____________
	**HOW WELL**			
**11.**	Planned: If intervention adherence or fidelity was assessed, describe how and by whom, and if any strategies were used to maintain or improve fidelity, describe them.		Page 4 (Section 2.3)	_____________
**12.**	Actual: If intervention adherence or fidelity was assessed, describe the extent to which the intervention was delivered as planned.		N/A	_____________
